# Double take

**DOI:** 10.1186/s12915-015-0217-2

**Published:** 2015-12-22

**Authors:** Emma Saxon

**Affiliations:** BMC Biology, BioMed Central, 236 Gray’s Inn Road, London, WC1X 8HB UK

## Abstract

Zebrafish are able to regenerate various organs and tissues after damage or amputation. To understand better the genetic controls of this process, the authors of this study investigated the expression of two genes previously implicated in fin regeneration using semi-quantitative RT-PCR, at three time points after fin amputation (T1, T2, and T3 in Fig. 1, corresponding to the initiation, middle, and end of fin regeneration, respectively). Briefly, the RT-PCR procedure involved isolating messenger RNA (mRNA) from a matched amount of zebrafish cells from the site of fin regeneration at the three time points, and using primers specific to each gene to selectively detect mRNA as an indicator of gene expression levels. The authors used total genomic DNA isolated from zebrafish cells as a positive control, and no RNA or DNA template as a negative control. They found that Gene 1 was only expressed early on in the process, while Gene 2 expression gradually increased during fin regeneration, reaching a peak of expression toward the end of the process. This provides some detailed information that could be useful in elucidating the function of these genes in fin regeneration.

## Comment

**Fig. 1:**
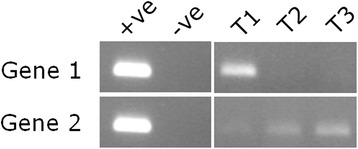
Gene expression profiles of Gene 1 and Gene 2 at the initation (T1), middle (T2), and end (T3) of fin regeneration after amputation, as measured by reverse-transcriptase polymerase chain reaction (RT-PCR). Gene 1 is expressed early on in the regeneration process, while Gene 2 is expressed towards the end. Zebrafish genomic DNA and no template were used in positive and negative control RT-PCR reactions, respectively

Robust controls are crucial in scientific studies, but may not always be appropriate or adequate. In Fig. 1, the same set of control images was used for RT-PCR expression studies of both genes. This means that only one set of primers, either for Gene 1 or Gene 2, was used as a positive control for the ability to amplify their target sequence in zebrafish genomic DNA. In other words, if the primers used in the control were specific to Gene 1, then it is impossible to tell whether the primers designed to investigate Gene 2 expression successfully amplified Gene 2: particularly important in this case, as detection of Gene 2 mRNA, and therefore Gene 2 expression, seems to be fairly low.

The authors could also have measured mRNA levels for a constantly expressed protein in all cells, such as actin, throughout fin regeneration as a further control. This would show whether the three RNA pools from the different time points contained equal amounts of RNA, and were equally free of contaminants that could interfere with the RT-PCR reaction, confirming real differences observed in Gene 1 or Gene 2 expression level across the time points.

To further understand the genetic control of fin regeneration, the authors could have taken additional mRNA samples before amputation and after the regeneration process was fully complete. This would have established whether Genes 1 and 2 were also expressed at either of these time points, and thus whether they are involved in cellular processes other than regeneration. This information could provide a more accurate picture of the genetic control of fin regeneration; for example, if Gene 1 was also expressed before and after regeneration, this would indicate that it is switched off during the later stages of fin regeneration, rather than being selectively switched on at initiation.

